# Therapeutic effect and study of human umbilical cord blood mononuclear cells in patients with ischaemic bowel disease

**DOI:** 10.1038/s41598-024-56720-z

**Published:** 2024-03-13

**Authors:** Xiaoxiao Cai, Yonghao Li, Fengyu Gao, Bilal Muhammad, Hongli Yang

**Affiliations:** 1 Shandong Provincial Maternal and Child Health Care Hospital Affiliated to Qingdao University, No. 238 Jingshi East Road, Jinan, Shandong China; 2 Department of Gastroenterology, The First Affiliated Hospital of Shandong First Medical University & Shandong Provincial Qianfoshan Hospital, No. 16766 Jingshi Road, Jinan, Shandong China; 3 Graduate Department of Shandong First Medical University & Shandong Academy of Medical Sciences, No. 6699 Qingdao Road, Jinan, Shandong China; 4 Department of General Surgery, The First Affiliated Hospital of Shandong First Medical University & Shandong Provincial Qianfoshan Hospital, No. 16766 Jingshi Road, Jinan, Shandong China

**Keywords:** Ischaemic bowel disease, Umbilical cord blood, Umbilical cord blood mononuclear cells, Interventional therapy, Stem-cell differentiation, Stem cells

## Abstract

Ischaemic bowel disease (ICBD) is a group of intestinal ischaemia syndromes caused by various aetiologies of reduced intestinal blood flow or vascular occlusion. ICBD can present as abdominal pain, bloody stool, and diarrhoea. This disease often occurs in middle-aged and elderly individuals with cardiovascular and cerebrovascular diseases. The incidence of ischaemic bowel disease has been increasing for decades, and it is difficult to diagnose, resulting in rapid disease progression and a high mortality rate. Therefore, fully understanding this disease, improving the diagnosis rate of this disease, and finding appropriate treatment methods are urgently needed to improve the condition and prognosis of patients. Umbilical cord blood stem cells are accessible, have weak immunogenicity, and have various biological functions, such as angiogenesis, inflammation and immune regulation. Many studies have confirmed that cord blood stem cells can relieve ischaemia, and these cells have attracted tremendous amounts of attention in regenerative medicine in recent years. In this paper, we discuss the clinical characteristics of ICBD, analyse the characteristics of human umbilical cord blood mononuclear cells (HUCB-MNCs), and use its to treat ischaemic bowel disease. Additionally, we compare the clinical manifestations and related indicators before and after treatment to evaluate the efficacy and safety of these methods.

## Introduction

**Figure 1:**
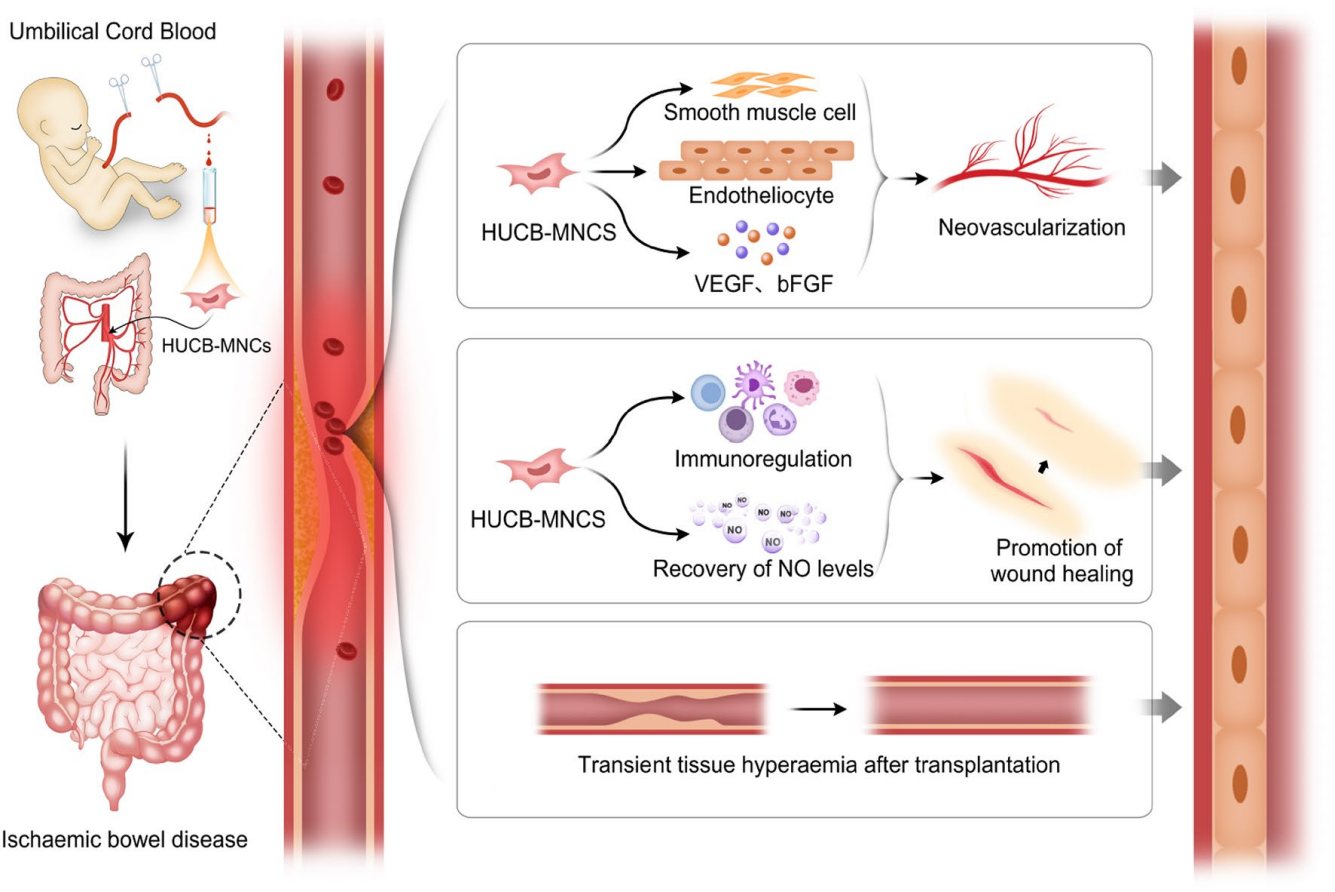
Mechanism of umbilical cord blood mononuclear cells in treatment of ICBD.

Ischaemic bowel disease (IBD) is a series of intestinal ischaemic syndromes due to an insufficient blood supply to the intestine caused by intestinal ischaemia and hypoxia^1^. The factors leading to this disease can be divided into acute mesenteric ischaemia (AMI), chronic mesenteric ischaemia (CMI) and ischaemic colitis (IC)^2^. The incidence of IC is the highest, reaching 22.9 per 100,000 people^3^, followed by acute mesenteric ischaemia. Several studies have reported that the total incidence of AMI in all patients hospitalized for acute surgery ranges from 0.09 to 0.2%^4^, and the overall incidence of chronic mesenteric ischaemia is approximately 1 per 100,000 people^5^; therefore, these cases are relatively rare. Elderly individuals with congestive heart failure, arrhythmia, myocardial infarction, diabetes and other diseases are at high risk^6^. The early clinical symptoms of ICBD are not typical, and the onset of abdominal pain, diarrhoea, and bloody stool are common. It is difficult to diagnose, and the misdiagnosis rate is as high as 60%^7^ (Fig. 1).

At present, this kind of disease is mainly treated by internal medicine. IC is mostly a self-limiting disease, and the symptoms of mild patients can be relieved by rest and diet adjustment and without the need for excessive interventions^8^. However, AMI has long been considered an inevitable and highly fatal disease. Despite the rapid progress in medicine, the incidence of AMI and overall mortality rate are still extremely high. Several reports have shown that the clinical mortality rate of AMI is still as high as 50–80% after active treatment^9^. If CMI is not treated in time, the risk of progressing to AMI is high, and the prognosis is extremely poor. Based on the clinical characteristics of this disease, it is particularly important to make a correct judgement in a timely manner and choose effective treatment methods to quickly relieve the condition and improve the prognosis of patients. The current treatment methods include medical symptomatic treatment, surgery and vascular interventional therapy. The effectiveness of medical treatment is poor, the problem of vascular stenosis cannot be fundamentally solved, and the recurrence rate is high. The surgical risk is high, and the postoperative mortality rate is high. Vascular intervention is a new treatment method that has the advantages of low surgical risk and rapid treatment, but the rate of postoperative intravascular restenosis is high. The incidence of stent restenosis is reportedly as high as 33%, and the mortality rate after acute stent occlusion is 50%^10^. The long-term quality of life of patients is poor, so it is urgent to find an effective treatment for ischaemic bowel disease.

When the body is experiencing ischaemia, compensatory effects mainly depend on the establishment of collateral circulation. Therefore, we investigated how to treat this disease at the cellular level by administering exogenous growth factors and vascular endothelial cells to generate new blood vessels and collateral circulation at the ischaemic site. Stem cells can differentiate into a variety of cells, generate tissues and organs with different functions, and have strong self-renewal ability^11^. Bone marrow, peripheral blood and cord blood contain a variety of stem cells, among which the number of bone marrow and peripheral blood haematopoietic stem cells is low, and the risk of transplantation is high; thus, the clinical application of these cells is relatively limited^12^. However, cord blood stem cells have the advantages of easy access, no harm to the donor, and no immune rejection and have been widely used to treat various difficult and miscellaneous diseases^13^.

Human umbilical cord blood mononuclear cells, which are isolated from the umbilical cord blood of pregnant women after delivery, are mononuclear cells and the largest cells in the human body; these cells include a variety of stem cells, lymphocytes and monocytes^14^. HUCB-MNCs have a variety of biological functions, such as tissue and cell regeneration, anti-inflammatory effects, and immune regulation^14^. Umbilical cord blood mononuclear cells can differentiate into a variety of immune cells as needed and can expand into a large number of highly active immune cells in vitro. When they are reinfused into the human body, they are not susceptible to rejection or side effects^15^, which provides more accurate solutions for many major diseases. At present, many studies have applied umbilical cord blood mononuclear cells to limb ischaemia and cardiovascular and cerebrovascular ischaemia.

## Results

### Comparison of baseline data before treatment

#### Comparison of general data and clinical manifestations

There was no significant difference in the general data or clinical manifestations between the two groups before treatment (*P* > 0.05), as shown in Table 1.

**Table 1:** Comparison of general data and clinical manifestations.

General information	Control group	Experimental group	Z/χ^2^	*P*
Age	65 (57.5,72)	68 (59.5,73)	− 0.379	0.705
Gender	13:20	16:17	0.554	0.457
Abdominal pain	29 (87.9%)	25 (75.8%)	1.63	0.202
Diarrhoea	21 (63.6%)	20 (60.6%)	0.064	0.8
Haematochezia	27 (81.8%)	23 (69.7%)	1.32	0.251
Abdominal tenderness	28 (84.8%)	26 (78.8%)	0.407	0.523

Gender is male: female; diarrhoea was defined as loose stools more than 3 times a day.

#### Comparison of laboratory examination between the two groups before and after treatment

There was no significant difference in laboratory test results between the two groups before treatment (*P* > 0.05), as shown in Table 2.

**Table 2:** Comparison of laboratory test results.

Ancillary tests	Treatment	Control group	Experimental group	Z/t	*P*
WBC(*10^9/L)	Before	7.75 (5.89,9.84)	5.97 (4.84,7.19)	− 1.513	0.13
	After	7.05 (6.39,8.42)	4.77 (4.05,5.86)	− 5.656	< 0.001*
NEUT (%)	Before	0.71 ± 0.12	0.66 ± 0.12	1.733	0.088
	After	0.68 ± 0.01	0.59 ± 0.13	3.20	0.002
HB(g/L)	Before	120.9 ± 17.0	115.4 ± 22.3	1.126	0.264
	After	118.97 ± 18.18	117.18 ± 23.00	0.35	0.727*
PLT(*10^9/L)	Before	223 (192.5,257.5)	237 (196.5, 269.5)	− 0.794	0.427
	After	209 (177.5, 255)	234 (186.5, 283.5)	− 1.481	0.139
DD (mg/L)	Before	1.31 (0.64, 3.77)	1.03 (0.36,2.29)	− 1.501	0.133
	After	1.64 (0.62, 3.59)	0.38 (0.19,1.14)	− 3.591	< 0.001*
PCT (ng/ml)	Before	0.08 (0.04,0.15)	0.13 (0.06, 0.22)	− 1.677	0.094
	After	0.08 (0.04, 0.59)	0.06 (0.04,0.16)	− 2.06	0.039*

*In the experimental group, the difference before and after treatment was statistically significant, *P* < 0.05.

### Comparison of therapeutic effects after treatment

#### Comparison of clinical manifestations and total hospitalization days between the two groups after treatment

There were significant differences in clinical symptoms between the two groups before and 5 days after treatment (*P* < 0.05). The hospitalization time of the experimental group was 9.55 ± 3.12 days, that of the control group was 11.75 ± 3.63 days, and the hospitalization time of the experimental group was significantly shorter than that of the control group (*P* < 0.05). The number of patients with abdominal pain, diarrhoea, bloody stool and abdominal tenderness in the experimental group was significantly lower than that before treatment (*P* < 0.05). See Table 3 for details.

**Table 3:** Comparison of clinical manifestations and length of hospital stay after treatment.

Symptoms	Control group	Experimental group	χ^2^/t	*P*
Abdominal pain	19 (57.6%)	9 (27.3%)	6.20	0.013*
Diarrhoea	17 (51.5%)	5 (15.2%)	9.82	0.002*
Haematochezia	20 (60.6%)	12 (36.4%)	3.88	0.049*
Abdominal tenderness	20 (60.6%)	11 (33.3%)	4.93	0.026*
Length of hospital stay	11.76 ± 3.63	9.55 ± 3.12	2.65	0.01

*In the experimental group, the difference before and after treatment was statistically significant, *P* < 0.05.

#### Comparison of laboratory tests

After treatment, the WBC, NEUT, PCT and DD in the experimental group and the control group had decreased significantly more than those in the control group (*P* < 0.05). There was no significant difference in HB or PLT between the two groups.After treatment, WBC, HB, DD and PCT in the experimental group had significantly improved compared with those before treatment (*P* < 0.05, showing statistical differences), as detailed in Table 2.

#### Comparison of clinical efficacy

In the control group, treatment was markedly effective in 9 patients, effective in 14 patients, and ineffective in 10 patients, for a total effective response rate of 69.7%. In the experimental group, 15 patients had markedly effective treatment, 15 patients had effective treatment, and 3 patients had ineffective treatment, for a total effective rate of 90.9%. The difference was statistically significant (*P* < 0.05). See Table 4 for details.

**Table 4:** Comparison of clinical efficacy (%).

Group	Conspicuous effect	effective	No effect	Effective rate
Control group	9 (27.3)	14 (42.4)	10 (30.3)	69.7
Experimental group	15 (45.5)	15 (45.5)	3 (9.1)	90.9
χ^2^				4.70
P				0.03

### Follow-up

#### Comparison of clinical symptoms at 1/3/6 months after treatment

One month after treatment, the number of patients with abdominal pain, bloody stool and abdominal tenderness in the experimental group was significantly lower than that in the control group (*P* < 0.05). After 3 months of treatment, the number of patients with abdominal pain and bloody stool in the experimental group was significantly greater than that in the control group (*P* < 0.05). After 6 months of treatment, the number of patients with abdominal pain, bloody stool and abdominal tenderness improvement in the experimental group was significantly greater than that in the control group (*P* < 0.05), as detailed in Table 5.

**Table 5:** Comparison of clinical symptoms at 1/3/6 months after treatment.

Symptoms	Time	Control group	Experimental group	χ^2^	P
Abdominal pain	1 month	17 (51.5)	10 (30.3)	3.07	0.08
	3 months	18 (54.5)	37 (21.2)	7.79	0.005
	6 months	16 (48.5)	4 (12.1)	10.33	0.001
Diarrhoea	1 month	15 (45.4)	9 (27.3)	2.36	0.125
	3 months	6 (18.2)	5 (5.2)	0.11	0.741
	6 months	3 (9.1)	3 (9.1)	0	1
Haematochezia	1 month	19 (57.6)	9 (27.3)	6.20	0.013
	3 months	16 (48.5)	8 (24.2)	4.19	0.041
	6 months	12 (36.4)	4 (12.1)	5.28	0.022
Abdominal tenderness	1 month	18 (54.5)	10 (30.3)	3.97	0.046
	3 months	11 (33.3)	7 (21.2)	1.22	0.269
	6 months	9 (27.3)	2 (6.1)	5.35	0.021

#### Comparison of colonoscopy reexamination after treatment

Within 6 months after treatment, 15 patients in the control group underwent colonoscopy; 8 of them had colonoscopy findings that were better than before, 4 had no significant change, and 2 were worse than before. In the experimental group, 11 patients underwent colonoscopy reexamination; 10 of them had colonscopy findings that were better than before, and 1 was worse than before. There was a significant difference in the effective rate between the two groups (*P* < 0.05). Table 6 for details.

**Table 6:** Comparison of colonoscopy reexamination results within 6 months after treatment.

	Cure	No effect	Aggravation
Control group	8 (53.3)	4 (26.7)	3 (20)
Experimental group	10 (90.0)	0 (0)	1 (9.1)
χ^2^			4.21
P			0.04

### Comparison of cord blood mononuclear cells before and after treatment

#### Comparison map of colonoscopy

**Figure 2:**
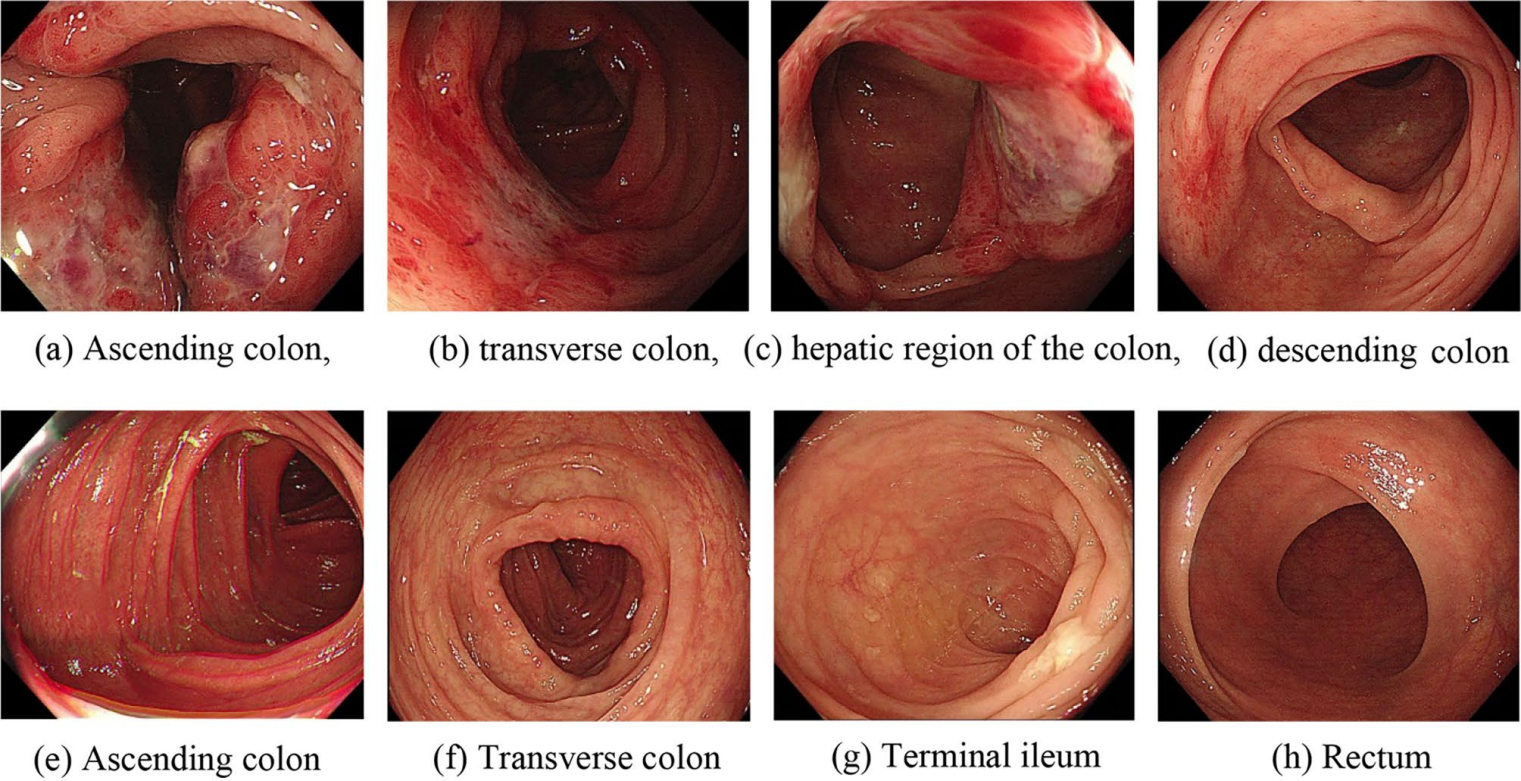
Comparison of colonoscopies before and after cord blood single-cell therapy for ICBD.

Figure 2a–d: Before treatment, multiple ulcers in the intestinal tract were observed via endoscopy; these ulcers were distributed along the longitudinal axis and covered with white fur-like material, and the surrounding mucosa was obviously hyperaemic and oedematous. Figure 2e–h: Colonoscopy reexamination 2 months after HUBC treatment showed smooth intestinal mucosa and clear peripheral vascular texture.

#### Contrast-enhanced mesangial contrast images (Fig. 3)

**Figure 3:**
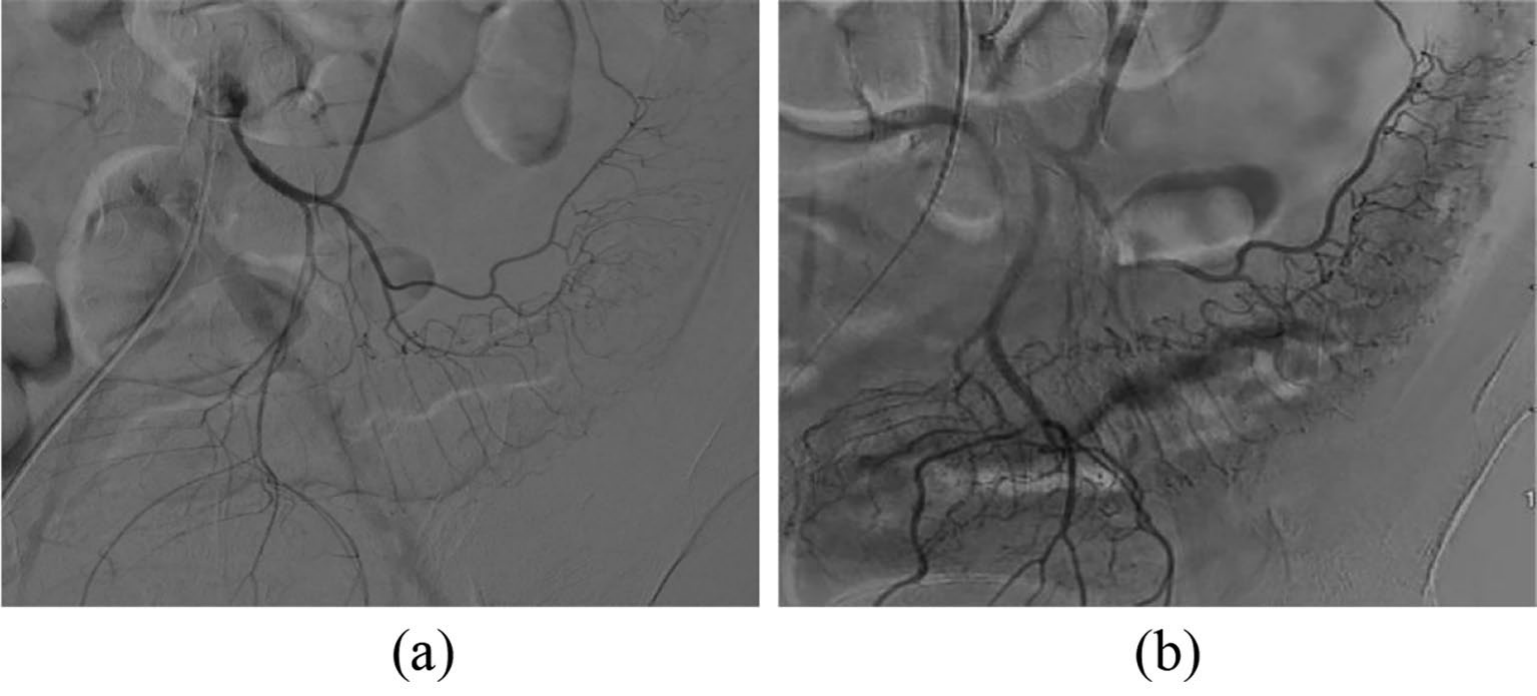
Comparison of inferior mesenteric artery angiography before and after treatment in the experimental group (**a**) Angiography before treatment showed thin and sparse blood vessels throughout the colon. (**b**) Three months after treatment with umbilical cord blood mononuclear cells, angiography showed that the mesenteric vessels were enlarged and that the blood flow had improved. (**a**) Angiography of the inferior mesenteric artery before treatment. (**b**) Posttreatment inferior mesenteric angiography

### Adverse reactions

We strictly monitored patients' vital signs, liver and kidney function and electrocardiogram before and after treatment, and used electrocardiogram monitoring during treatment to ensure patients' life safety. There were no treatment-related adverse events, new tumours or deaths in the two groups during the treatment or follow-up.

## Discussion

There are different causes of different types of ischaemic bowel disease. Ischaemia in AMI often occurs in the small intestine, and the most common cause is acute intestinal ischaemia caused by sudden interruption of the blood supply caused by detachment of emboli from the superior mesenteric artery^16^. The majority of CMI cases are caused by a gradual reduction in the intestinal blood supply due to atherosclerosis of the intestinal vessels, and mesenteric vascular ischaemia occurs slowly. The ischaemic site of IC occurs in the colon, and patients often have a vascular nonocclusive ischaemic injury due to vasospasm and contraction that is caused by factors such as hypovolemia or drugs. This type of gastrointestinal injury is the most common form of gastrointestinal ischaemic injury and accounts for more than half of gastrointestinal ischaemic cases^17^. A reduction in blood flow caused by vascular occlusion leads to intestinal ischaemia and hypoxia, intestinal vascular endothelial cell damage, vascular contracture, platelet aggregation, vascular embolism formation and many inflammatory reactions^18^. When intravascular thrombosis occurs, the fibrinolytic system is activated, and fibrin is degraded. The plasma D-dimer concentration is a site of action in the fibrinolysis process, and an increase in D-dimer indicates that the blood is in a hypercoagulable state. At present, many studies at home and abroad have confirmed that the detection of plasma D-dimer has high diagnostic and prognostic value for ischaemic bowel disease^19^. Platelets (PLTs) can undergo coagulation, haemostasis and repair of damaged blood vessels. When the vessel wall is damaged, PLTs quickly adhere to the surface of damaged endothelial cells and undergo a series of complex reactions to repair endothelial cells^20^. When thrombosis or inflammatory reactions occur in the body, the PLT count usually increases. When microcirculation disorders occur in the body, the intestinal blood supply is affected, and tissue ischaemia and hypoxia can result in the production of a large number of oxygen free radicals, which can destroy the intestinal mucosal barrier^21^. At this time, intestinal peristalsis is slowed, which can cause bacterial reproduction and intestinal flora translocation. White blood cells (WBCs) function in chemotaxis and deformation. When invaded by bacteria, WBCs can penetrate the vascular wall and quickly reach the invasion site, engulfing and phagocytosing the bacteria^22^. It has been reported that more than 90% of patients with ischaemic bowel disease have abnormally elevated WBCs^23^. The neutrophil percentage (NEUT) is the proportion of neutrophils among white blood cells, and this ratio is highly important for diagnosing inflammatory infection and determining the degree of infection. When extensive tissue necrosis and severe bacterial infections occur, the percentage of neutrophils is often high^24^. Procalcitonin (PCT) is a known marker of bacterial inflammation, and its concentration in healthy people is very low^25^. PCT has the advantages of a short operation time, good stability, and difficulty in degrading. Its level can well reflect the severity of infection^26^. Therefore, real-time dynamic detection of the PCT concentration in blood is beneficial for observing therapeutic efficacy. Ayten et al. reported that the PCT level in the experimental group was significantly greater than that in the control group when intestinal ischaemia occurred, which was inferred to be related to the inflammatory response caused by intestinal ischaemia^27^. Our findings were also consistent with the related literature. At the same time, bloody stools cause a decrease in haemoglobin, which occurs in some patients. In conclusion, we used the above indicators as the criteria for evaluating whether the treatment was effective when the experiment was designed.

In the early stage of intestinal ischaemia, the intestinal tract in the lesion area still retains a certain extent of vitality. Moreover, if active treatment is performed to improve the intestinal blood supply, further exacerbation of ischaemia can be avoided^28^. Different types of ICBD have different treatment focuses. Patients with clinically suspected ischaemic bowel disease should be fasted, undergo gastrointestinal decompression, and receive oxygen and nutritional support immediately^29^. While actively controlling the primary disease, certain treatments, such as correcting intestinal hypoperfusion caused by hypotension, shock, or cardiac insufficiency and controlling blood pressure and blood glucose, should be initiated^29^. Patients with thrombosis should be treated with anticoagulant and thrombolytic therapy as soon as possible but only after undergoing a bleeding risk assessment. Drug therapy is suitable only for mild patients, and surgical or vascular intervention is often needed for moderate and severe patients^30^. Interventional therapy involves opening occluded blood vessels or removing emboli through guidewires, catheters, balloons, stents and other devices^31^. This treatment has the advantages of safety, less trauma, quick recovery, and fewer complications and has been routinely used to treat ischaemic diseases^32^**.** However, interventional therapy itself is a kind of trauma to blood vessels and may cause a local inflammatory response in blood vessels after trauma. The incidence of postoperative restenosis is high. Surgical treatment is usually adopted for patients with severe ischaemic bowel disease, especially when the patient has intestinal infarction or acute peritonitis signs requiring immediate laparotomy^33^. The surgical wound area is large, healing is slow, and postoperative mortality is very high. ICBD progresses rapidly, and the existing treatment methods have their own advantages and disadvantages. The improper selection of treatment can easily delay the initiation of appropriate treatment, causing irreversible intestinal injury and critical problems.

Cell therapy, which involves the use of primary cells, cell lines and various stem cells, has been a promising method for the treatment of difficult diseases in recent years^34^. Through transplantation and local infusion of autologous or allogeneic cells, cells and/or biologically active molecules are used to repair or enhance the biological function of damaged tissues^35^. In mammals, stem cells include embryonic stem cells and adult stem cells. Human embryonic stem cells were initially used for reproduction through in vitro fertilization in the 1990s and have since been used to investigate potential roles in regenerative medicine^36^. However, because this process involves the destruction of human embryos, related research is ethically and politically controversial^37^. Umbilical cord blood stem cells are a type of adult stem cell that has unique advantages compared with bone marrow and peripheral blood haematopoietic stem cells: (1) Stem cells in the umbilical cord are easy to collect, harmless to the donor, have less ethical controversy, and have stronger proliferation and differentiation ability^38^. (2) UCB is rich in haematopoietic stem cells, and its concentration and purity are 10–20 times greater than those of bone marrow haematopoietic stem cells^39^. (3) Also, the T and B lymphocytes in cord blood are naive because they have no contact with their own substances, their immune function is not mature, and their antigenicity is low^40^. (4) Umbilical cord blood cells are protected by the placental barrier and are rarely contaminated^41^. (5) Cord blood cells can be cryopreserved for 15 years or more, 60–90% of viable cells can be recovered after resuscitation, and the preparation time for transplantation is short, which is suitable for patients with rapid disease progression^42^. Therefore, cord blood stem cells are an ideal source for stem cell transplantation.

When an ischaemic event occurs, the local microenvironment of ischaemic tissue changes, and “attracts” exogenous stem cells to precisely home toward the ischaemic tissue and interact with vascular endothelial cells in the ischaemic tissue. This cell‒cell interaction is mediated by E-selectin adhesion molecules^43^. In ischaemic tissue, E-selectin is highly expressed on activated endothelial cells and acts as a “docking site” to anchor stem cells expressing corresponding E-selectin ligands in the blood circulation, thereby inducing tissue repair and angiogenesis^44^. For the treatment of ischaemic bowel disease, some research directions include promoting angiogenesis and increasing blood supply at the ischaemic site. Stem cell transplantation can increase blood flow at the ischaemic site by inducing neovascularization and collateral circulation.

Umbilical cord blood mononuclear cells include haematopoietic stem cells (HSCs), mesenchymal stem cells (MSCs), endothelial progenitor cells (EPCs), lymphocytes, and monocytes^45^. HSCs are specialized stem cells and one of the most abundant stem cells in UCB; these cells can differentiate into cells of the blood and immune systems^46^. At present, HSCS are mainly used to treat various refractory haematological diseases.

MSCs can be induced to differentiate into vascular endothelial progenitor cells, vascular smooth muscle cells, nerve cells, cardiomyocytes, osteoblasts and hepatocytes, etc^47^. MSCs have been gradually used in the clinical treatment of cardiovascular and cerebrovascular ischaemia, limb ischaemia, Parkinson's disease, liver cirrhosis and other diseases and have achieved good clinical effects^48^. Intramuscular injection of placental-derived MSCs has been shown to have greater therapeutic efficacy than bone marrow or adipose tissue-derived MSCs in a mouse hindlimb ischaemia model^49^. MSCs can support haematopoiesis, express adhesion molecules, interact with haematopoietic cells, regulate the adhesion between haematopoietic cells and stromal cells, and play an important role in haematopoietic cell implantation and homing^50^. MSCs promote angiogenesis mainly through two pathways. On the one hand, MSCs differentiate into vascular endothelial cells and smooth muscle cells to directly form new blood vessels^51^. On the other hand, through its powerful paracrine effect, microglia secrete angiogenic factors such as basic fibroblast growth factor (bFGF), vascular endothelial growth factor (VEGF) and angiopoietin to indirectly promote angiogenesis^43,52^. B-FGF is a polypeptide that can promote angiogenesis. Its role is to promote the mitosis of vascular endothelial cells (VECs) and induce VECs to move to the ischaemic site to produce VEGF^53^. VEGF is a functional glycoprotein with high biological activity that can induce VEC migration, proliferation and differentiation to form new blood vessels^54^. Several studies have confirmed that VEGF levels in the transplanted area are significantly increased after local injection of umbilical cord blood mononuclear cells into an animal model of bilateral lower limb ischaemia^55^, and these results further confirmed the role of MSCs in promoting angiogenesis. In addition, MSCs can also participate in the body’s immune response via two possible mechanisms. One is that they participate in immune regulation by inhibiting the proliferation of T cells and stimulating the proliferation of local endothelial cells to promote intestinal healing in a synergistic manner^56^. On the other hand, changes in the microbiome may be involved. The gut microbiome plays an important role in stimulating intestinal integrity and local immune responses. Recent studies have shown that the diversity of intestinal microbes is also significantly increased in animals treated with MSCs^57^.

EPCs are the precursor cells of VECs. The main functions of EPCs include inhibiting the inflammatory response of the vascular wall, reducing intimal hyperplasia to promote the repair of the injured endothelium, and preventing the narrowing of blood vessels^58^. EPCs play an important role in ischaemic tissue. On the one hand, EPCs differentiate into new VECs by themselves and participate in angiogenesis, and this type of angiogenesis does not rely on the existing vascular system^59^. On the other hand, these cells rely on their own paracrine effect and secrete some growth factors and other proangiogenic substances to indirectly participate in angiogenesis^60^.

In summary, we speculate that the mechanism of cord blood mononuclear cells in the treatment of ischaemic bowel disease may be as follows:

1. Transient tissue hyperaemia after transplantation. In our study, we found that clinical manifestations such as abdominal pain and abdominal tenderness were significantly relieved 5 days after treatment in most patients after infusion of HUCB-MNCs through the mesenteric artery, but neovascularization and new collateral circulation had not yet formed. This improvement in symptoms may be related to vasodilatation, tissue congestion and temporary blood supply to the ischaemic site after the infusion of cells. Tissue hyperaemia quickly alleviates the symptoms of ischaemia for a short period of time, and the patient’s clinical symptoms are alleviated; however, this tissue response gradually weakens with time after transplantation.
2. Promotion of angiogenesis. We found that the clinical symptoms of the patients who received umbilical cord blood mononuclear cell transplantation were significantly less common than those of the control group, which was considered to be related to the formation of neovascularization and new collateral circulation.
3. Promotion of wound healing. The degree of bloody stool in patients treated with HUCB-MNCs was significantly lower than that in the control group and before treatment. On the one hand, these findings were related to the improvement in ischaemia, and on the other hand, they may be related to the mechanism through which HUCB-MNCs promote wound healing. Wound healing is actually achieved by the growth and expansion of some cells whose repair functions involve mainly stem cells from the wound, wound periphery, or blood^61^. Serum nitric oxide (NO) is an antioxidant secreted by vascular endothelial cells and is a key mediator of tissue repair. It can remove superoxide free radicals, regulate vascular tone, relax blood vessels, prevent coagulation and eliminate thrombosis^62^. During ischaemia and hypoxia, the levels of endogenous nitric oxide synthase (NOS) inhibitors increase, and NO synthesis decreases. Treatment with HUCB-MNCs may reduce the level of NOS inhibitors and promote the recovery of NO levels, thereby accelerating wound healing and vasodilation^63^. In this experiment, DD in the experimental group was lower than that in the control group after treatment, which further verified this claim.Collagen is a structural protein of the extracellular matrix that functions to provide strength and integrity to the tissue matrix and plays an extremely important role in wound healing^63^. It has been confirmed that wound contraction and epithelial cell formation occur earlier in the injured areas of skin treated with HUCB-MNCs, and marked vascular and fibrous hyperplasia can be observed around the wound. Moreover, the weight of the granulation tissue increased more obviously^64^. In addition, compared with those of dermal fibroblasts, the collagen synthesis and bFGF and VEGF levels of stem cells are markedly greater, indicating that HUCB-MNCs can also change the tissue microenvironment and promote wound healing and tissue repair by secreting soluble factors^65^.
4. Regulation of immune and anti-inflammatory effects and reduce inflammatory cell infiltration into the wound. HUCB-MNCs secrete growth factors, such as hepatocyte growth factor, insulin-like growth factor, VEGF, bFGF and anti-inflammatory cytokines, such as IL-10^66^, which have antiapoptotic and antinecroptotic effects on ischaemic and injured cells. McDonald et al. demonstrated that UCB and special types of UCB cells, such as EPCs and monocytes, can regulate peripheral and central immune responses^67^. In this way, the immune inflammatory response around the ischaemic injury site is reduced. Our trial showed that the WBC, NEUT and PCT counts of the experimental group were better than those of the treatment group after treatment, which also indicated that the inflammatory response was inhibited after HUCB-MNCs treatment, which was consistent with the findings of previous reports.

Umbilical cord blood mononuclear cells are involved in compensatory vascular reconstruction in the ischaemic region and can improve and restore blood flow at the ischaemic site. A number of studies have confirmed that umbilical cord blood mononuclear cells are safe and effective in the treatment of ischaemic diseases. Cho et al.^68^ found that HUCB-MNC transplantation can activate the angiogenesis signalling pathway mediated by interleukin-8 (IL-8), promote angiogenesis in the injured area, and reduce the area of cerebral infarction. Huang et al.^69^ found that intra-arterial infusion of HUCB-MNCs in stroke rats can improve regional intravascular blood flow, vascular reactivity and vascular function and reduce the infarct volume caused by ischemia. In several studies, umbilical cord blood mononuclear cells have been injected into rats with lower limb ischaemic diseases, and the experimental observations revealed that the subjective symptoms and objective indicators of the rats were significantly improved after treatment. A small amount of collateral circulation was found at the transplantation site, and no deaths, tumours, retinal detachments or other sequelae occurred during the study, suggesting that umbilical cord blood mononuclear cells promote angiogenesis. The safety of the treatment is also illustrated.

## Conclusion

Umbilical cord blood mononuclear cells have very broad application prospects and have achieved good clinical efficacy in the treatment of lower limb ischaemic diseases. The use of umbilical cord blood mononuclear cells promotes angiogenesis and wound healing, improves blood circulation, improves ischaemia, and opens up new ideas for the treatment of ischaemic diseases. At present, there is no specific treatment for ischaemic bowel disease, and conservative medical treatment alone has a high recurrence rate and mortality. This study used multiple or multiple transplantations of umbilical cord blood mononuclear cells to treat ischaemic bowel disease. The results showed that patients treated with umbilical cord blood mononuclear cells had better clinical symptoms and related auxiliary examinations than did those treated with drugs alone. The clinical remission rate in the experimental group was better than that in the control group, and no treatment-related adverse reactions or complications occurred, which further supports the benefit of umbilical cord blood mononuclear cells in the treatment of ischaemic bowel disease. It was also confirmed that HUCB-MNCs have the advantages of weak immunogenicity and decreased immune rejection. It is relatively easy to obtain umbilical cord blood mononuclear cells. If umbilical cord blood mononuclear cells can be widely used in the improvement and treatment of ischaemic bowel disease under the premise of ensuring safety, this approach will be useful for the majority of patients.

## Limitations of this study

First, although the incidence of ischaemic bowel disease is increasing annually, the overall incidence is still low, and the sample size of this study was small. Thus, there is a high possibility of error in the results. If more patients are enrolled from multiple centres, the statistical results will be more convincing. In addition, both the experimental group and the control group were treated in this study. In principle, placebo treatment was more appropriate for the control group, but due to ethical restrictions, both groups were treated. Second, because colonoscopy and mesenteric angiography are invasive procedures and relatively expensive, some patients have a poor compliance toward these two methods. Therefore, this study failed to include comprehensive and regular dynamic follow-up data on the vascular and tissue levels of the two groups of patients, and only the subjective indicators were followed up, which is highly likely to cause errors. Although our results showed that the experimental group was superior to the control group in terms of comprehensive response rate, clinical remission severity and follow-up results, to ensure effectiveness and safety, it is necessary to conduct a relatively comprehensive comparison of patients with umbilical cord blood mononuclear cells before routine clinical treatment for ischaemic bowel disease to further confirm our conclusions.

## Methods

Sixty-six patients with ischaemic bowel disease diagnosed and hospitalized in our hospital between January 2019 and June 2022 were randomly divided into a control group (n = 33) and an experimental group (n = 33). In both groups, the patients were fasted and given fluid infusion, and medications for haemostasis, anti-infection, vasodilatation, intestinal mucosa repair, intestinal flora regulation and other medical treatments, were also given. In the experimental group, umbilical cord blood mononuclear cells were infused through the superior and/or inferior mesenteric artery, and growth hormone was given to promote stem cell differentiation after the operation. The two groups were reexamined on the 5th day after treatment, and the changes in clinical manifestations and related laboratory indicators before and after the treatment were compared. The adverse reactions related to and during the treatment were recorded, and the efficacy and safety of cord blood stem cell therapy were preliminarily analysed. The patients were followed up at 1, 3, and 6 months after treatment to compare and evaluate the long-term therapeutic effect of the two treatment methods.

### Diagnostic criteria

The diagnostic criterion for acute mesenteric ischaemia (AMI) was from the “Acute mesenteric ischaemia: Guidelines of the World Society of Emergency Surgery”^70^. The clinical manifestations were sudden severe abdominal pain, fever, bloody stool, acidosis and organ failure, which are highly suggestive of the disease. Severe abdominal pain is incompatible with mild abdominal signs. CTA generally shows partial or complete obstruction of the mesenteric blood vessels and can be used to diagnose the disease.

The diagnostic criteria for chronic mesenteric ischaemia (CMI) were based on the Chronic Mesenteric Ischaemia Clinical Practice Guideline from the “Chronic Mesenteric Ischaemia Clinical Practice Guideline from the Society for Vascular Surgery Journal of Vascular Surgery (2020)”^31^. The clinical manifestations were recurrent abdominal pain and a non-fixed pain location, and postprandial abdominal pain, fear of eating, and weight loss were typical triad symptoms. CTA was the first diagnostic imaging test, and mesenteric arteriography indicated that vascular stenosis > 70% could be diagnosed.

The diagnostic criteria for ischaemic colitis (IC) were based on the “Ischaemic colitis: Uncertainty in diagnosis, pathophysiology and management”^71^. The clinical manifestations are unexplained abdominal pain, mainly spasmodic pain in the left lower abdomen accompanied by the impulse to defecate, and bloody stool usually occurring within 24 h. The symptoms are not typical, other diseases that may cause intestinal infection should be excluded during diagnosis, and the patient's history, imaging, endoscopic findings and histopathology should be combined.

### Inclusion criteria

① No age, no sex;
② Those who met the above diagnosis;
③ Patients who were willing to provide signed informed consent for treatment and cooperate with treatment.

### Exclusion criteria

① Patients with severe necrosis of the intestinal wall caused by severe ischaemic bowel disease requiring surgical treatment;
② Patients with other inflammatory bowel diseases;
③ Patients with serious disease of other organs;
④ Patients who cannot cooperate and are unwilling to provide informed consent;
⑤ Patients who missed visits or had automatic discharge.

### Sources of cord blood mononuclear cells

The umbilical cord blood mononuclear cells used in this study were all sourced from Shandong Qilu Cell Therapy Engineering Technology Co., LTD. (Cell size: 20 ml, 2.0 × 10^8^/case, number of live cells ≥ 90%), and all cells were guaranteed to be negative for bacteria, fungi, viruses and bacterial endotoxins.

### Procedure of umbilical blood mononuclear cell infusion

1. The umbilical cord blood mononuclear cells were separated and stored by professionals in special test tubes and placed in standard containers at 2–8 °C, after which a small number of cells were counted, and bacterial culture and various tests were performed.
2. In the intervention room, the patient's superior and/or lower mesenteric artery angiographs were first recorded and videotaped by senior physicians with more than 5 years of working experience to observe the distribution of the patient's mesenteric blood vessels, after which mononuclear cells were injected. The speed of cell input was not fast, and the control was approximately 2 ml/min. After the operation, the puncture point was compressed to stop bleeding for 8–24 h.
3. Growth hormone was given via a subcutaneous injection after surgery, the patient’s vital signs were detected, routine medical treatment was continued, and treatment-related adverse reactions were recorded over time.

### Observation indicators

1. Clinical manifestations: abdominal pain, defecation frequency, bloody stool, and abdominal tenderness;
2. Laboratory indicators: white blood cell (WBC) count, neutrophil percentage (NEUT), haemoglobin (HB), platelet (PLT), D-dimer (DD), and procalcitonin (PCT) count; routine stool test; liver and kidney function; and electrocardiogram.
3. Colonoscopy findings: mucosal congestion, oedema, ulceration, and clear vascular texture;

#### Efficacy criteria

##### Cure

The clinical symptoms and signs had disappeared, the results of routine stool examination were negative for occult blood, the frequency of stool was less than 3 times a day, and the laboratory test results were within the normal range.

##### Conclusion

Compared with previous results, the occult blood test results from a routine stool examination were negative or weakly positive, and the clinical manifestations and laboratory test results had improved.

##### Ineffective

The clinical manifestations were unchanged or even aggravated compared with before treatment, and the laboratory indices were not improved or even aggravated.

#### Record of adverse reactions

Anaphylaxis, liver and kidney damage and new electrocardiogram changes were recorded during the treatment.

### Statistical analysis

The data were imported into SPSS 27.0 software for statistical analysis. The measurement data were tested for normality and homogeneity of variance. The normally distributed data were tested by two-sample t tests and are presented in $\overline{x}$ ± s. Nonnormally distributed data were analysed by the nonparametric rank sum test and are expressed as M (P25, P75). Categorical data were analysed by the chi-square test, and count data are expressed as n (%). A difference of 0.05 was considered to indicate statistical significance.

### Flowchart of the clinical trial (Fig. 4)

**Figure 4:**
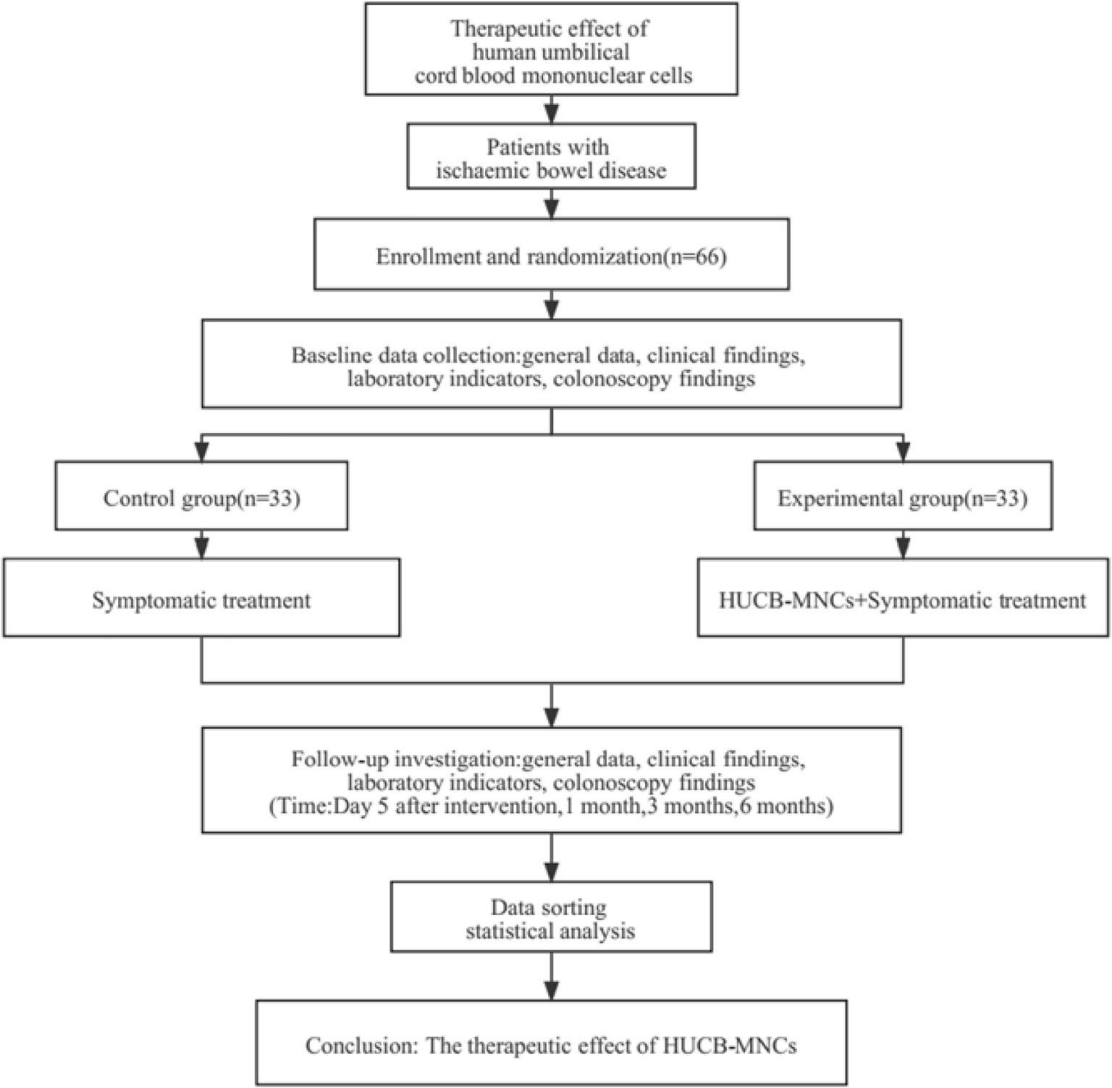
Clinical trial design process.

#### Statement

This study was recognized by the First Affiliated Hospital of Shandong First Medical University, and the licensing committee approved the experiments. All the experiments were performed in accordance with the relevant guidelines and regulations.

## Data Availability

The data that support the findings of this study are available from the corresponding author upon reasonable request.
